# Methodological support for forensic science in the USA and Ukraine: A comparative study

**DOI:** 10.1016/j.fsisyn.2024.100571

**Published:** 2025-01-04

**Authors:** Nataliia Martynenko

**Affiliations:** Ph.D. in the Field of Public Management and Administration, Associate Professor of the Department of Political Sciences and Law, Kyiv National University of Construction and Architecture, 31, Povitroflotsky Avenue, Kyiv, 03037, Ukraine

**Keywords:** Forensic expert activity, Scientific research, Methodological support, Forensic examination methods, Forensic expert opinion, Standardization, International cooperation

## Abstract

The significant potential of proper methodological support in solving key tasks of forensic expert activity is noted. The procedure for certification and state registration of forensic examination methods introduced in Ukraine is analyzed. The composition and procedure for the activities of the advisory bodies of the Ministry of Justice of Ukraine which determine the relevance and priority of forensic examination research are investigated. The author identifies the areas of concern in the methodological support of forensic institutions, the addressing of which relates to the activities of the State in terms of legal support of forensics at the legislative and departmental regulatory levels.

The requirements to the registration of research results by grantees of the US National Institute of Justice are clarified. The article compares research costs, access to scientific results, and promising research topics in the field of forensic science in the United States and Ukraine. Certain ways of solving the identified problems related to the methodological support of forensic expert activity were proposed, taking into account the specifics of the legal system of Ukraine and the experience of the National Institute of Justice in the United States. The author emphasizes the need for further development and strengthening of cooperation between forensic institutions and international organizations in the field of standardization.

## Highlights

• Elimination of competition between expert methods will ensure the weight of forensic expert opinions.
• Establishment of a single methodological body will help eliminate expert conflicts.
• Proper methodological support for forensic examination will help improve the scientific level, quality and timing of forensic examinations.
• The development of a unified methodological approach to method representation will contribute to the development of national legislation on forensic examination.
• Modern high-tech methods of examining forensic objects require evaluation by expert laboratories around the world.
• Cross-country comparison of research topics, costs of methods development, and access to scientific results will contribute to harmonizing forensic cooperation.

## 1 Introduction

At the current stage of development of forensic expert activity in Ukraine, a closer attention is paid to ensuring the credibility of the expert opinion as one of the types of evidence in legal proceedings. This is due to a significant increase in requests for the use of specialized knowledge and, accordingly, an inevitable increase in the expert workload, and a tighter timeframe for conducting the necessary investigations. However, a decline in forensic examination quality is unacceptable, since the cost of an expert error can be very high, with unreliable results leading to irreparable consequences for a person's fate [1,2]. Forensic expertise exerts a significant impact on the beliefs of the decision maker [3].

The solution to this issue is not to increase the staff of experts, but to raise forensic institutions’ performance by optimizing their activities. Achieving this goal is complicated by departmental disunity, a growing number of experts who are not employees of forensic institutions, and the lack of a unified methodological approach to conducting examinations [4].

An important area of regulating forensic institutions’ activities, as well as the work of forensic experts who are not employees of specialized institutions, is methodological support which includes, in particular, the development of forensic examination methods, their certification and state registration. A forensic examination method is the result of a scientific work comprising a system of research techniques used to carry out a specific expert task [5].

Methodological support concerns the following three aspects: firstly, the formation at the state level of a unified approach to procedures for the development, testing, implementation, use and optimization of forensic investigation methods and techniques (macro level); secondly, the unification at the local level of a separate branch of scientific knowledge about the standards for conducting highly specialized investigations (meso level); thirdly, defining the limits of the expert's autonomy in choosing methods and techniques when performing an examination assignment in criminal proceedings [6]. It is essential for methodological support to algorithmize the expert's actions, reduce subjectivity, and introduce new expert technologies that enable obtaining more accurate and reliable results with fewer resources and in a shorter time [6].

Ensuring a high degree of scientific validity of forensic research methods is of particular importance. The relevant international experience should be studied in order to use it in a wide range of measures aimed to improve the quality of investigations and shorten their time-limits.

The website of the European Network of Forensic Science Institutes (ENFSI) offers the best forensic practice manuals and guides funded by the European Commission: *Animal, Plant and Soil*; *Digital Imaging*; *DNA*; *Drugs*; *Explosives*; *Fingerprints*; *Firearms/GSR*; *Fire and Explosions Investigation*; *Forensic Information Technology*; *Forensic Speech and Audio Analysis*; *Handwriting*; *Paint, Glass & Taggants*; *Road Accident Analysis*; *Scene of Crime*; *Textile and Hair*; *Evaluative Reporting*; *Proficiency Tests and Collaborative Exercises* [7].

The main purpose of the openly available practical guidelines for conducting expert research on the ENFSI website is to improve the quality of forensic institutions throughout Europe and thereby promote the standardization of forensic processes and cross-border cooperation between countries, the use of a harmonized methodology and the elaboration of comparative results, which will facilitate the recognition of the outcomes of laboratory activities of all the ENFSI member states, as well as other interested countries.

ENFSI as a network of over seventy European forensic institutes has a strong interest in understanding scientific and technological trends that will contribute to creation of new methods needed to address the issues that the forensic community will deal with in the coming years [8].

New tools and technologies are constantly developed within various scientific disciplines, which leads to increased diversity and fragmentation of forensic science [9]. The complexity of scientific information, the processes of scientific knowledge integration and differentiation, the emergence of new objects that did not exist or were transformed by the use of innovative technologies, encourage the creation of new methods as systems of recommendations and mandatory rules for forensic experts that determine their research designs and strategies [10]. The main task is to verify the reliability and accuracy of methods and techniques, the creation of which was facilitated by scientific and technological innovations [11,12].

In forensic research projects, there is no clear distinction between the research focused on management decision-making and that focused on science and/or justice [13]. More should be done at the government level to support fundamental research in the field of forensic science aimed at creating new tools and methods [14].

Joint conducting of scientific research by forensic laboratories will help reduce their workload [[15], [16], [17]]. Successful interaction between public forensic laboratories and corporate or academic institutions contributes to the development of innovative technologies and methods [18]. Conventional methods of closed testing are at odds with the aspirations of the criminal justice system which needs accurate and transparent forensic methods [19]. Forensic laboratories should understand the importance of sharing data on their research [18]. The development, adoption and implementation of new tools and technologies in forensic science is facilitated by research results publication [18]. In order to ensure the transparency of forensic examinations, attention has recently been focused on the study of standardization practices [3]. A standardized approach to the classification of quality issues is needed [20].

Despite a long history of developing the above problems and a multitude of scientific sources, a number of issues of both theoretical and practical nature remain unresolved in Ukraine. The purpose of this article is to identify and study the existing problems related to the methodological support of forensic expert activity, and to provide some ways of addressing them, taking into account the specifics of the legal system of Ukraine, as well as the relevant experience of the National Institute of Justice in the United States.

## 2 Methods

The methodological basis of the study was formed by philosophical, general scientific, and special legal methods of cognition, used in a comprehensive way in accordance with the principle of legal pluralism. In the course of the study, the author made an extensive use of the dialectical method of cognition and the method of legislative analysis. Among the general scientific methods of cognition, the formal legal method and information processing techniques of analysis, synthesis, analogy, induction, and deduction were of special importance for studying the provisions of laws and regulations. The functional and legal method was used to highlight the powers of the scientific councils of specialized forensic institutions, the Scientific Advisory and Methodological Council on Forensic Expertise, and the Coordinating Council on Forensic Expertise at the Ministry of Justice of Ukraine (hereinafter - the Coordination Council) in matters of certification and state registration of forensic examination methods. The statistical method helped to estimate the actual costs of research and development. The method of legal forecasting made it possible to determine the directions of further development of scientific views on the methodological support of forensic expert activity. The use of the tools of the comparative legal method which utilizes synchronic and diachronic methods made it possible to form a rational system of coordinates for further development of the methodological support for forensic expert activity.

## 3 Results and discussion

### 3.1 Procedure for certification and state registration of forensic examination methods in Ukraine

According to Art. 8 of the Law of Ukraine “On Forensic Expertise”, organization of the scientific and methodological support for forensic activities and the organizational and managerial principles of state specialized forensic institutions are assigned to ministries and other state bodies, managing the state specialized institutions that carry out forensic activities [21]. Ministries and other government agencies monitor in due course compliance with the law by forensic institutions under their jurisdiction. The methodological support of the activities of forensic experts who are not employees of state specialized forensic institutions is entrusted to the Ministry of Justice of Ukraine.

As specified by Art. 8 of the Law of Ukraine “On Forensic Examination”, the methods of conducting forensic examinations (except for forensic medical and forensic psychiatric examinations) are subject to certification and state registration in accordance with the procedure established by the Cabinet of Ministers of Ukraine [21].

In compliance with the Procedure for Certification and State Registration of Forensic Examination Methods [5] (hereinafter - the Procedure), the methods are developed by state specialized forensic institutions, namely: research institutions of forensic examinations of the Ministry of Justice of Ukraine and the Ministry of Health of Ukraine; forensic services of the Ministry of Internal Affairs of Ukraine, the Ministry of Defense of Ukraine, the Security Service of Ukraine and the State Border Guard Service of Ukraine, as well as by forensic experts who are not employees of these institutions. Leading experts in the relevant fields of knowledge may also be involved in methods development with their consent.

The Procedure defines the course of actions for certification and state registration of forensic examination methods (except for forensic medical and forensic psychiatric ones); amendments to the methods that have passed certification and state registration; and discontinuation of the use of methods. The law also establishes that methods previously implemented in expert practice are considered to have passed certification and are subject to state registration.

Certification of methods consists of reviewing the report on the scientific work on methods development and the validation of methods. Reviewing a report on the conducted scientific work involves preparation of a written conclusion by leading experts in the respective fields of knowledge who did not participate in the method development about its relevance and novelty, taking into account modern advances in science and technology, as well as about the possibility of using the method in expert practice. The method validation consists in preparation of a statement by specialists of specialized forensic institutions who did not participate in method development analizing the validity of the method, as well as its effectiveness and efficiency for performing expert tasks.

The method is submitted for state registration after the scientific councils of specialized forensic institutions review the results of its certification and make a decision to recommend the method for implementation in expert practice. Methods submitted for state registration are reviewed by the Coordinating Council. After the Coordinating Council makes a decision on granting state registration, the method is assigned a corresponding registration code, and therefore is subject to inclusion in the Register of Forensic Examination Methods; otherwise it is returned to the developer.

The Coordinating Council on Forensic Expertise at the Ministry of Justice is a permanent advisory body established to develop common approaches to the organization of forensic activities and to address the most important issues of forensics development that are of interdepartmental nature [22]. Expenses related to the activities of the Coordinating Council are covered within the allocations for the maintenance of the Ministry of Justice of Ukraine. The organizational and technical support of the Coordinating Council is entrusted to the Ministry of Justice of Ukraine.

The Coordination Council is composed of representatives of ministries and other state bodies whose jurisdiction includes state specialized forensic institutions; heads or deputy heads of state specialized institutions; forensic experts who are not employees of state specialized institutions with at least five years of experience in forensics and not brought to disciplinary responsibility during the last year, in the number of no more than five persons [22]. The personal composition of the Coordinating Council is approved by an order of the Ministry of Justice of Ukraine upon submission of forensic expert candidacies by the concerned state bodies and public organizations (no more than one person from a public organization of forensic experts). The members of the Coordinating Council work on a voluntary basis.

The Coordination Council, in accordance with the tasks assigned to it:

1) considers issues related to: state registration of forensic examination methods (except for forensic medical and forensic psychiatric expertise) submitted by the Ministry of Justice for consideration by the Coordination Council, or refusal to register them; amendments to forensic examination methods that have been certified and registered; termination of the use of forensic examination methods and development of methods to replace the existing ones in cases provided for by law; priority of forensic examination research; creation of new research methods and introduction of new types of forensic examination by the subjects of forensic activity; application of scientific and methodological recommendations and methods of forensic examination; the use of scientific, technical and reference literature, as well as software products during forensic examinations; formation of the Register of Forensic Examination Methods; establishment and development of international cooperation and exchange of scientific and technical achievements in the field of forensic examination, etc.;
2) approves scientific and methodological recommendations (except for forensic medical and forensic psychiatric expertise) which include unified approaches to determining the objects of examination for the relevant types (subtypes) of forensic examinations, the main tasks and an indicative list of examination issues to be resolved [22,23].

Immediately it should be noted that the problem is in the fact that the scientific communities reviewing reports on research works, from which expert examination methods are derived, are the scientific councils of specialized forensic institutions and the Coordinating Council that examines all the documents to make a final decision on registration [24]. The Procedure for Certification and State Registration of Forensic Examination Methods specifies the reviewing of the research reports on methods and their validation techniques, while the structure and content of the methods proper are neglected, the validation procedure also being out of focus [24].

By the decision of the Coordinating Council on Forensic Expertise at the Ministry of Justice of Ukraine dated 28.01.2021, the list of scientific and technical literature and software products recommended for use in expert practice included a report on the research work *Updating the methodological recommendations “Expert method development: content, structure, design”* [25]. This research work unifies the content and structure of expert methods in the form of documents, the procedure for their execution in accordance with the provisions of the amended versions of international standards of quality management systems adopted in Ukraine [26].

The state registration of methods is carried out by the Ministry of Justice of Ukraine which determines the organizational and methodological principles of maintaining the Register of Forensic Examination Methods - an official electronic database that creates an information collection of certified forensic examination methods that are recommended for implementation in expert practice [27]. The holder of the Register of Forensic Examination Methods is the Ministry of Justice of Ukraine. The Register of Forensic Examination Methods contains the following information: method registration code, type of examination, method title, name of method developer, the year of method development, its amendment year, the year of method discontinuation, and dates of decisions: on state registration, on state registration of amendments, and on discontinuation of the method use [28].

It should be pointed out that the current Law of Ukraine “On Forensic Examination” makes no mention of the Register of Forensic Examination Methods. Moreover, the Register itself does not provide a possibility of access to the methods in electronic form.

In this regard, of interest is the information set forth in a scientific paper on a survey of forensic experts’ view of the Register of Forensic Examination Methods: it needs to be improved - 70 experts (40.7 %); it is of great practical importance - 64 experts (37.2 %); it does not contain a summary of expert methods - 34 experts (19.8 %); other - 4 experts (2.3 %) [29].

By the decision of the Coordinating Council on Forensic Expertise at the Ministry of Justice of Ukraine dated 28.01.2021, for the first time it was recommended to include in the Register of Forensic Examination Methods under the relevant registration codes 12 methods that had been certified and recommended by the decision of the scientific councils of the State Scientific Research Forensic Center of the Ministry of Internal Affairs of Ukraine, the National Academy of Internal Affairs, the Ukrainian Scientific Research Institute of Special Equipment and Forensic Expertise of the Security Service of Ukraine. Prior to this, the state registration of methods developed by the forensic research institutions of the Ministry of Justice of Ukraine had been carried out.

According to the analytical report of the Ministry of Justice of Ukraine, as of December 31, 2023, 1363 methods were registered (Table 1) which require regular updating.

**Table 1:** Information on methods registered in the Register of Forensic Examination Methods.

Areas of expert research	Number of methods
examination of materials, substances and products	417
engineering and technical expertise	180
technical examination of documents	129
trace evidence examination	109
economic expertise	104
handwriting and linguistic expertise	84
psychological examination	81
examination of weapons	44
biological expertise	41
examination of sound- and video-recording	35
commodity expertise	27
explosive technical expertise	26
comprehensive examination	24
art history expertise	17
intellectual property expertise	16
photographic and portrait expertise	15
military expertise	6
examination of special technical means of covert information acquisition	5
veterinary expertise	2
historical and archeological examination	1

Among the existing problems related to the methodological support of forensic activities in Ukraine are the following:

1) lack of a unified methodological approach to method representation – the existing methods were created at different times, by different agencies, have different structures and, accordingly, differ in content;
2) absence of a single methodological body that would include a certain unit getting representatives of other expert agencies involved in study of methods and decision-making on their registration, which would eliminate conflicts occurring in expert practice;
3) competition of expert methods, given the adversarial system of legal proceedings - when different forensic institutions, possibly at different times, have developed methods of examination of the same object that differ to some extent from each other, which casts doubt on the weight of forensic experts' conclusions: if opposing conclusions can be scientifically subsqtantiated, the reliability of the results obtained by a forensic expert is questionable;
4) lack of access to registered methods used in practice by experts, investigators, prosecutors, courts and defense attorneys, which makes it impossible to evaluate expert opinions, as there are cases when experts of one forensic institution know nothing about the methodology developed by forensic experts of another agency;
5) lack of an effective interdepartmental mechanism for providing the relevant judicial and law enforcement agencies with information about the existence of certain methods or the possibility of forensic examinations in general [24].

### 3.2 Requirements for research results documentation by recipients of grants from the National Institute of justice in the United States

The NIJ expects grantees to create scientific products, such as peer-reviewed journal articles, review papers, software, databases, and patents which will be the main deliverables. In addition, awardees must submit a Final Research Report on scientific finding for Non-forensic Research or a Technical Summary on scientific finding for Forensic Science Research no later than the last day of the grant project period. Grantees should expect that the final report will be made public in whole or in part.

The Final Research Report and Technical Summary should be well-developed, concise and suitable for publication. There is no specific page limit, however too long Final Research Reports may be returned.

The Final Research Report and Technical Summary should be written in such a way that their content is understandable for a wide audience, including researchers, practitioners, policy makers, and prepared with the intention of open access posting in the NCJRS Virtual Library.

There are established requirements for the design and structure of the Final Research Report and Technical Summary. Sections of the Final Research Report that should be cumulative and prepared with regard to public dissemination are specified [31]. It is also determined what the Technical Summary should include [31].

### 3.3 The Scientific Advisory and Methodological Council on Forensic Examination at the Ministry of Justice of Ukraine

**Fig. 1:**
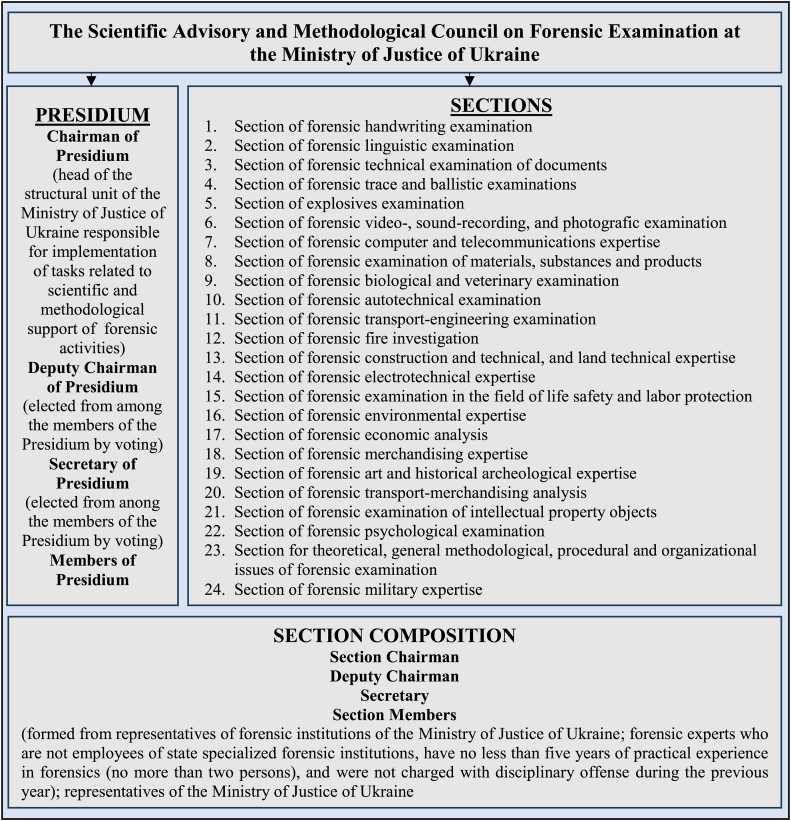
The structure of the Scientific Advisory and Methodological Council on Forensic Examination at the Ministry of Justice of Ukraine.

The Scientific Advisory and Methodological Council on Forensic Examination (hereinafter referred to as SACFE) is an advisory body established by the Ministry of Justice of Ukraine to improve the quality of research work, promote the effectiveness of scientific developments and the quality of expert activities of research forensic institutions (hereinafter referred to as RFI), which fall under the Ministry of Justice of Ukraine, and of forensic experts who are not employees of state specialized forensic institutions [32]. SACFE determines the priority areas of scientific research in the field of forensic examination and provides proposals on the forms and methods of their implementation and introduction into expert practice; it assesses the prospects for the development of scientific research in the field of forensic examination, its relevance and novelty, etc. It should be noted that SACFE operates on a voluntary basis. The structure of SACFE is shown in Fig. 1.

Compiled by the author based on [[32], [33]].

The Chairman of a section is elected exclusively from among its members who are employees of forensic institutions; the Deputy Chairman and the Secretary of the section are elected from among its members. Specialists from the central executive body which has jurisdiction over state specialized forensic institutions, as well as from scientific and educational institutions of other state bodies may also participate in the work of the sections. According to para. 3.4 of the Regulation on the Scientific Advisory and Methodological Council on Forensic Examination at the Ministry of Justice of Ukraine, “in order to include certified forensic experts who are not employees of state specialized forensic institutions in the section, an announcement on section formation shall be posted on the website of the Ministry of Justice of Ukraine, which contains the requirements to section members, an address for submitting proposals and the deadline for submission” [33].

It should be noted that the current version of the Regulation on the Scientific Advisory and Methodological Council on Forensic Examination at the Ministry of Justice of Ukraine does not contain information on the deadlines for posting an announcement on section formation, nor does it specify in which part of the website of the Ministry of Justice of Ukraine the announcement on section formation is posted.

The main form of activity of SACFE sections is meetings convened twice a year (fall and spring). During the meetings of SACFE sections, proposals of forensic institutions and forensic experts who are not employees of specialized forensic institutions as to research works to be included in the thematic plan are considered; the progress of research works is discussed; the results of completed research works are reviewed and accepted; issues of their validation and implementation in expert practice are resolved; proposals for amendments to the Register of Forensic Methods and the List of Recommended Scientific, Technical and Reference Literature used in conducting forensic examinations are prepared; proposals for amendments and supplements to the methods implemented in expert practice are submitted for consideration by the Coordinating Council on Forensic Expertise at the Ministry of Justice of Ukraine; issues related to the introduction of new types of forensic examinations and methods of their conduct are considered; and more.

Meetings of SACFE Presidium are held when necessary, but at least once a year. At the meetings of SACFE Presidium, the decisions of the sections are considered, disputes are resolved, etc. The decisions of the sections are advisory in nature, while the decisions of the Presidium of SACFE are binding for RFI of the Ministry of Justice of Ukraine.

### 3.4 Research implementation at research forensic institutions of the Ministry of Justice of Ukraine

According to Art. 15 of the Law of Ukraine “On Forensic Examination”, “forensic institutions’ carrying out research works on scientific developments related to organization and conduct of forensic examinations is financed by the State Budget of Ukraine” [21]. The Ministry of Justice of Ukraine, in accordance with the tasks assigned to it, organizes, as set by law, expert support for justice and research in the field of forensic examination; coordinates the activities of ministries and other central executive authorities on forensic examination development; organizes the work of the Coordinating Council and SAСFE, etc. [34].

The Report on the results of the management efficiency audit of the forensic research institutions of the Ministry of Justice of Ukraine, which has financial implications for the state budget, approved by the decision of the Accounting Chamber dated 23.12.2022 No. 28-1 (hereinafter - the Accounting Chamber Report) states that “paragraph 3 of Procedure No. 372/5 stipulates that the Ministry of Justice of Ukraine is the customer of scientific products and manages the scientific activities of RFI within the powers defined by the current legislation. Exclusive property rights to scientific work results as intellectual property belong to the state represented by the Ministry of Justice of Ukraine” [35]. Procedure No. 372/5, to which the Accounting Chamber refers, is a departmental regulatory act and is not publicly available.

Based on the results of the research work audit of RFI of the Ministry of Justice of Ukraine, the Accounting Chamber concludes in its Report that the Ministry of Justice of Ukraine, while implementing the function of directing and controlling the activities of RFI, did not take the appropriate measures to prevent the risks of uneconomical use of budget funds in the area of “Development of Forensic Methodology”, understatement of the work cost, and non-recording of intangible assets (property rights) to the balance sheet [35].

The initial cost of research and development work performed by RFI consists of actual labor costs and payroll accruals for scientists and experts involved in this work. No other costs were included in the calculation. Starting in 2022, actual research and development spending increased significantly, as shown in Table 2.

**Table 2:** Expenditures and performance indicators in the area of the budget funds use“Development of Forensic Methodology” for the period 2020–2023.

	2020	2021	2022	2023
Total (USD ths.)	1398.5	1449.6	1753.3	1478.3
including: general fund (USD ths.);	1398.5	1449.6	1753.3	1478.3
special fund (USD ths.)	–	–	–	–
Number of developments (pcs.)	129	118	128	110
Number of completed developments (pcs.)	50	49	45	64
Average cost of 1 development (USD ths.)	10.8	12.3	13.7	13.4
Share of completed developments in the total number of developments (%)	38.8	41.5	35.2	58.2

Let us take a closer look at the costs of developing research techniques and methods in the field of forensic science, which are implemented in forensic practice, taking into account modern advances in science and technology, using the example of 2022 (Table 3).

**Table 3:** Expenditures and performance indicators in the area of the budget funds use “Development of Forensic Methodology” in 2022.

	planned	amended plan	actual	deviation of amended plan from planned (±)	deviation of actual from amended plan (=/-)
Total (USD ths.)	1980.0	1782.0	1753.3	−198.0	−28.7
including: general fund (USD ths.);	1980.0	1782.0	1753.3	−198.0	−28.7
special fund (USD ths.)	–	–	–	–	–
Number of developments (pcs.)	128.0	128.0	128.0	0.0	0.0
Number of completed developments (pcs.)	77.0	62.0	45.0	−15.0	−17.0
Average cost of 1 development (USD ths.)	15.5	13.9	13.7	−1.6	−0.2
Share of completed developments in the total number of developments (%)	60.2	48.4	35.2	−11.8	−13.2

The dynamics of the indicators is stipulated by the introduction of new types of examinations which require the use of new techniques and, accordingly, creation of new methodologies [44].

The Passport of the Budget program for 2022 provides for the general fund of USD 1980.1 thousand for the area of “Development of Forensic Methodology” [41,45].

In accordance with the thematic plan of research and development, 128 research projects were carried out in 2022, of which 45 were completed. Based on the results of the completed developments, 21 methods, 15 methodological recommendations, 2 manuals and 7 draft Ukrainian State Standards (DSTU) were prepared.

According to the Results of the performance evaluation of the Budget program “Conducting Forensic Examination and Development of Forensic Examination Methodology” for 2022, the reasons for the decrease in completed developments, the terms of which were extended by the decision of SACFE at the Ministry of Justice of Ukraine until the end of 2023, are as follows: the armed aggression of the Russian Federation; changes in the legislation of Ukraine; the use of new types of weapons and military equipment by the Armed Forces of Ukraine; the change of research and development chief scientists; introduction of new expert specialties in the areas of “Molecular Genetic Research” and “Thermal-Engineering Research”; amendments to the Register of Expertise Methods (the outdated methods and those authored by representatives of the Russian Federation and the Republic of Belarus, which are referenced by RFI developers in almost all expert specialties, were excluded) and to the Lists of Recommended Scientific, Technical and Reference Literature used in forensic examinations (exclusion of references to Russian sources, etc.) [38].

The Report on the implementation of the Passport of the Budget program for 2022 provides a statement of reasons for the deviation of cash expenditures (budget loans) in the area of budget funds use from the amounts approved in the Budget program Passport: “the amount of expenditures for the development of the methodology for conducting forensic examinations decreased by USD 226.71 thousand, including:

- in accordance with the Resolution of the Cabinet of Ministers of Ukraine No. 401 “On Allocation of Funds to the Reserve Fund of the State Budget” dated 01.04.2022 [46], the expenses for developing methodology of forensic examinations were reduced by USD 198.0 thousand;
- due to the reduction of the amount of the compulsory state social insurance contribution for employees with disabilities, and due to the excess, in some cases, of the maximum accrual base of the single contribution (the maximum amount of income of the insured person per month established by law, on which the single contribution is charged), there is a saving of USD 28.7 thousand” [41,47].

The Passport of the Budget program for 2023 in the area of “Development of Forensic Methodology” in the general fund provides for USD 1478.3 thousand for 110 developments, the average cost of 1 development is USD 13.4 thousand [42,48].

According to the Thematic plan of research work for 2023, it was planned to develop methods and guidelines for 110 topics; and for 2024 - for 86 topics, of which 52 topics were launched in 2019–2023 [49].

Most of the research proposed for 2024 is related to the armed aggression of the Russian Federation. The list of 34 topics proposed for research starting in 2024 includes the following developments:

- a methodology for conducting forensic economic examinations of the accrual and payment of financial support and additional remunerations to the military personnel of the Armed Forces of Ukraine;
- a methodology for postmortem forensic psychological examination in criminal proceedings concerning the suicide of the servicemen of the Armed Forces and other military formations of Ukraine during martial law;
- methodological recommendations for the use of unmanned aerial vehicles in military research;
- methodological recommendations for conducting comprehensive military and transport engineering research to assess the actions of combat vehicle drivers in traffic incidents that occurred under martial law;
- methodological recommendations for conducting comprehensive examinations related to intellectual property objects;
- methodological recommendations for expert examination of humanitarian goods for military purposes and dual use;
- methodological recommendations on forensic examination of goods in cases of illegal trafficking of humanitarian aid;
- methodological recommendations for determining the damage caused to the environment as a result of the armed conflict in Ukraine;
- methodological recommendations for evaluating the damaging factors of ammunition explosions and detecting the direction (location) from which they were used;
- a practical guide for conducting expert studies of the UAV “Shahed – 136”, of the Russian Federation marking “Geran-2”, and more [49].

The Thematic plans of research works include the following information: research work title, executing institutions, chief scientists, and deadlines. The website of the Ministry of Justice of Ukraine does not contain information on the costs of developing methods and guidelines by topics. Information about the essence, purpose, and stages of research is not disclosed either.

### 3.5 Grant support for research in the field of forensic science in the United States

In the United States, almost all funds for research in the field of forensic science are channelled through the Department of Justice. For the most part, research in the field of forensic science is financed by the National Institute of Justice (NIJ) and the Federal Bureau of Investigation [50]. Despite the fact that the United States does not have a universal authority over forensic science providers, all levels of government that have forensic science providers under their jurisdiction receive federal funding [51].

Since 2009, NIJ has invested nearly $300 million in its forensic science research program and funded more than 660 forensic science development projects, which makes it a leader in the development of expert support for justice. In September 2023, NIJ announced $16-million funding to support 33 projects under the FY 2023 Forensic Science for Criminal Justice Research and Development Program. Through this program, NIJ constantly improves the reliability, accuracy, and efficiency of forensic science, and upgrades its methods, which ultimately contributes to the administration of justice [52]. In order to expand the collaboration and capabilities of forensic laboratories, NIJ informs forensic laboratory heads about research trends, technologies, partnership opportunities, and Federal government efforts [53].

The procedure used by NIJ to determine which proposals should be funded is quite lengthy. First, all proposals are reviewed by independent expert committees composed of both researchers and practitioners. The committee members read each proposal, assess the technical merit and policy relevance of the proposed research, and meet to discuss their assessments against the terms and conditions set forth in the solicitation. The committees’ evaluations and any accompanying reports by NIJ staff are forwarded to NIJ Director. All final decisions on grant awards are made by the Assistant Attorney General (AAG) [54].

In 2023, NIJ grants were directed to research on the following topics:

Developmental Validation of a Novel Multi-analyte Recovery Method for Trace Biological Samples, $494,268;

Time Since Deposition Signatures for Touch DNA Evidence, $389,621;

Optimizing Analytical Parameters for Detection of Chronic and Single Dose Drug Exposure in Forensic Hair Analysis, $431,892;

An Image Analysis Framework for Objective Color Interpretation of Seized Drug Tests, $392,196;

Quantifying the Strength of Support in Fingerprint Casework Comparisons, $609,185;

Accuracy, Efficacy, and Reproducibility of Muzzle-to-Target Distance Determination using Gunshot Residue, $872,282;

Analytical Challenges with Proliferating THC Analogues, $726,360;

Mechanics of Retinal Hemorrhage in Abusive Head Trauma, $604,409, and more [52].

It should be noted that each topic is publicly available: the essence and purpose, expected theoretical and practical results, performers, and stages of the research.

NIJ typically releases funding opportunities beginning in late fall. In 2024, NIJ plans to fund the following research:

Research and Evaluation for the Testing and Interpretation of Physical Evidence in Publicly Funded Forensic Laboratories;

Research on the Abuse, Neglect, and Financial Exploitation of Older Adults;

Research on the Impact of Technologies for Forensic Science Applications, etc. [55]. However, the proposed topics may change.

NIJ requires that grant applicants submit a data archiving plan which specifies how research will be documented, managed, and prepared for archiving, as well as where the data is to be archived after the project is completed. Archiving of research works by grant recipients ensures that federally funded data is preserved, transparent, and accessible for discovery, reuse, reproduction, replication, and continuation of research by other scientists.

Forensic science projects are archived in the National Archive of Criminal Justice Data (NACJD) or in repositories appropriate to the field of study, subject to the NIJ-approved Data Archiving Plan. Regardless of the primary data repository used, all NIJ-funded forensic science projects must provide NACJD with information about level of research together with a project abstract and a link to a publicly accessible data location, ideally with a DOI or other permanent reference [56,57].

NIJ recommends submitting products resulting from NIJ-funded work (journal articles, published final reports, etc.) to the National Criminal Justice Reference Service (NCJRS) Virtual Library, which already has 235,000 resources [58]. The Research Forensic Library, a collaboration between Florida International University and the National Institute of Justice, offers thousands of articles and reports in all areas of forensic science in open access [59].

### 3.6 Standardization as a tool for improving forensic examination quality and objectification

International cooperation in the field of standardization, particularly in forensic science, is associated with the activities of the International Organization for Standardization (ISO), an international non-profit, independent, non-governmental organization whose members include representatives of national standardization bodies from 172 countries [60].

As part of ISO's activities, an international project committee ISO/SC 272 “Forensic Sciences” was established in 2012, which in 2016 turned into a standing technical committee under the same name. The scope of its activities is standardization and guidance in the field of forensic science. ISO/TC 272 “Forensic Sciences” comprises 27 full members and 21 observers [61].

To date, three standards that fall within the scope of ISO/TC 272 “Forensic Sciences” have been brought out [62]:

– ISO 18385-2016 “Minimizing the risk of human DNA contamination in products used to collect, store and analyze biological material for forensic purposes - Requirements” was published in 2016 and last revised and validated in 2022 [63];
– ISO 21043–1:2018 “Forensic Sciences – Part 1: Terms and definitions” was published in 2018 and is expected to be replaced by ISO/DIS 21043-1 in the coming months [64];
– ISO 21043–2:2018 “Forensic Sciences – Part 2: Recognition, recording, collection, transport and storage of items” was published in 2018 and is expected to be replaced by ISO/DIS 21043-2 in the next few months [65].

ISO standards are developed by experts who work in a particular industry, understand and anticipate its problems and use standardization as a tool to create a level playing field.

Since the standards must meet the requirements of time, science and technology, they are regularly reviewed and updated every five years.

The three remaining parts of the 21043 series: Part 3, Analysis, Part 4, Interpretation, and Part 5, Reporting, are being developed at various stages of readiness [62].

Ukraine is an observer member of ISO/TC 272 “Forensic Sciences” [66]. The subject matter of the International Committee ISO/TC 272 was enshrined in the order of the national standardization body, whose functions are performed by the State Enterprise “Ukrainian Research and Training Center for Standardization, Certification and Quality”, No. 125 dated 07.05.2018 on the establishment of the Technical Committee for Standardization 192 “Forensic Sciences” (hereinafter – TC) [67]. The functions of the Secretariat of TC were assigned to Kyiv Scientific Research Institute of Forensic Expertise of the Ministry of Justice of Ukraine, and the order No. 175 of 19.06.2018 approved the Regulations on TC 192 “Forensic Sciences” [68]. TC 192 is composed of 23 collective members and 5 individual ones, including state expert research institutions of the Ministry of Justice of Ukraine, the Ministry of Internal Affairs of Ukraine, the Security Service of Ukraine, the State Customs Service of Ukraine, the Ministry of Health of Ukraine, and others.

TC 192 harmonized one international standard and two parts of another as regards: minimizing the risk of contamination of products used for the collection, storage, analysis of biological material for forensic purposes and human DNA; terminology and definitions in the forensic process; as well as the collection (detection, recording, seizure, transportation and storage) of objects of forensic significance during the inspection of crime scenes. In addition, TC 192 has developed 6 more national standards. Currently, work is underway to develop more than 24 national standards of Ukraine [68].

It should probably be noted that the classical legal systems that have developed historically over the centuries are the Anglo-American system, which includes the United States, and the Romano-Germanic system, which, given the legal foundation laid, includes Ukraine. Unification occurs through the convergence and harmonization of the legal systems of different states by borrowing relevant legal structures and smoothing out fundamental differences between the laws of legal regulation. The internationalization of law and process is manifested in the gradual convergence of different legal systems through their interaction and interpenetration in the course of international cooperation, and in the creation of common legal spaces within the framework of legal integration [69]. The list of sources for the unification of legal systems includes standards – international, regional, in particular European, and national, adopted by the relevant standardization organizations.

The American Society for Testing and Materials (ASTM) established Committee E30 on criminalistics in 1970 [70]. The 335-member Committee exercises jurisdiction over 71 forensics standards. E30 incorporates 5 technical subcommittees that regulate the standards. These standards still take the lead in all aspects of criminal science, including forensic examinations, digital and multimedia evidence, fire debris analysis, drug testing analysis, physical and digital evidence collection and preservation, and reporting of results.

In 2009, the world of forensics exploded with a joined report of the National Research Council (NRC) and the National Academies of Sciences (NAS) “Strengthening Forensic Science in the United States: A Path Forward” [42]. In 2014, taking into account the proposals for standardization, the National Institute of Standards and Technology (NIST) together with the Department of Justice (DOJ) established the Organization of Scientific Area Committees (OSACs) to strengthen the US forensics [71]. The OSAC mission is to promote the development of technically sound standards, expand the OSAC Registry with standards that have passed technical evaluation, and support the implementation of these standards by OSAC stakeholders and the forensic science community [72]. However, OSAC is not a government-recognized standard development organization.

Established in 2015, the Academy Standards Board (ASB) is a subsidiary of the American Academy of Forensic Sciences (AAFS) accredited by the American National Standards Institute (ANSI) as a standard development organization. By focusing on the development and implementation of standards, ASB promotes collection of reliable data, reducing bias in the justice system [73]. The United States is a full member of ISO/TC 272 “Forensic Sciences”. ANSI is an American member of ISO.

For the purposes of judicial proceedings, especially criminal proceedings, when conducting forensic examination, all experts should use only unified, standardized, scientifically sound and empirically tested methods [23].

Best practices, manuals, standard operating procedures, etc. within quality management systems should document ‘how’ the requirements of the standard will be met, while the national regulations and policies define ‘who’ should meet these requirements.

In order to improve the quality of forensic science, it is necessary to continue to develop and strengthen forensic institutions’ cooperation with international standardization organizations, both through joint work in ISO/TC 272 and by participating in various communication activities offered by regional networks of forensic laboratories around the world.

The variety of cooperation activities in the field of standardization, involving heads of forensic organizations, experts, specialists, and scientists from a majority of countries of the world, undoubtedly contributes to the development of all the participants.

### 3.7 Suggestions for Ukraine

In order to solve the problems related to the methodological support of forensic activities, taking into account the specifics of the legal system of Ukraine and the experience of the National Institute of Justice in the United States, it has become necessary to implement consistently a set of tasks:

1) to create a single body with the authority of providing methodological support for forensic activities that will analyze forensic examination methods and decide on their registration, which will eliminate expert conflicts;
2) to develop a unified methodological approach to the presentation of methods by setting forth in the relevant regulatory act the requirements to the structure and content of expert methods, which will facilitate the uniformity of their design, regardless of which agency develops a method;
3) to supplement the current Law of Ukraine “On Forensic Examination” with provisions on the purpose and structure of the Register of Forensic Examination Methods, the procedure for its maintenance, content and the use of information. To enshrine in the Law the issues of protecting information of the Register of Forensic Examination Methods, control over compliance with information protection requirements, and to determine the options for providing access to forensic methods for employees of state forensic institutions who are qualified as forensic experts, as well as for forensic experts who are not employees of these institutions;
4) to enshrine in the Law of Ukraine “On Forensic Examination” the requirement to publish information on the costs of methods and guidelines development, the essence, purpose, stages, expected theoretical and practical results by research topics on the websites of the relevant ministries and other state bodies that manage state specialized forensic institutions;
5) to develop and adopt a procedure for conducting research work by forensic institutions and by forensic experts who are not employees of these institutions which will determine the requirements for planning, conducting, and accepting research works, as well as testing and implementing their results; the procedure should be made publicly available. The following issues need to be addressed:
- exclusive property rights to research and development results as intellectual property;
- responsibility for the scientific level, organization and efficiency of research, for the quality and timely execution of research works;
- proper sequencing of research and development activities;
- defining the tasks of the research leader who prepares and conducts coordination activities with co-executors (if any); determines the content of tasks, deadlines and scope of work by stages for each executor; systematically monitors the timeliness and quality of tasks performed by each executor; summarizes, analyzes and evaluates the results obtained upon completion of the stages and upon completion of research on a selected topic, etc;
- planning of research work carried out in accordance with the main directions of implementation of the state scientific and technical policy in the field of forensic examination;
- conducting research work, which includes: selection of areas; theoretical and experimental research; generalization and evaluation of research results;
- determining the structure of the research report;
- acceptance of research results with the aim of their assessment as to: novelty and prospects of the proposed results, the use of domestic and foreign achievements of science and technology; patentability and competitiveness of the proposed scientific and research-technical solutions; compliance of the results with the technical requirements, technical specifications and other documents, etc;
- establishing certain results validation and implementation procedures;
6) to resolve at the legislative level the issues of creating a virtual library of methods, guidelines, scientific reports based on the results of research in the field of forensic science, and providing access to registered methods for their practical use by experts, as well as by investigators, prosecutors, courts and defense attorneys evaluating expert opinions.

Addressing the above theoretical and practical tasks will facilitate the improvement of the scientific level and completeness of expert tasks, which will have a positive impact on forensic examination quality and timing.

In fact, it will become possible to properly evaluate expert opinions as equal sources of evidence in the process of proving, which would guarantee compliance with the principle of adversarial proceedings.

In a democratically developed civil society, the state acts in the interests of society and its institutions, promotes the development and functioning of self-governing and self-regulatory capabilities [74]. The Law of Ukraine “On Forensic Expertise” contains provisions discriminatory against non-governmental forensic experts, which is why the country's non-governmental forensics has not been able to become a tool that promotes competition in the field of specialized knowledge and experience. Currently, Ukraine needs to develop a legislative act to establish a self-government system for forensic experts.

Due to the implementation of the proposed set of tasks, pursuing the goal of ensuring the best possible competitiveness of the parties and their dispositive right to freedom in providing evidence, the existing model of forensic support of justice which is not ideal due to corporate and political interests, including corruption and abuse of office, will undergo revision and transformation.

## 4 Conclusion

In summation of our study, it should be noted that there is an obvious need to introduce uniform mechanisms for conducting examinations by all forensic institutions, as well as by forensic experts who are not employees of these institutions, in order to ensure the uniformity of requirements, uniformity of methodological support, and uniformity of management principles and forensic activities results. The processes taking place at the present stage of forensic science development encourage elaboration, testing and practical application of modern high-tech methods of researching forensic objects, and necessitate the improvement of the quality of forensic activities through introduction, implementation and evaluation of new mechanisms and uniform requirements among national forensic institutions, forensic experts who are not employees of these institutions, and expert laboratories around the world.

Harmonization of the ‘rules of the game’ at the international and national levels will contribute to the development of international cooperation in order to carry out coordinated standardization activities. Legally regulated criteria for the implementation of standardization mechanisms in forensic science are the basis which allows developing and making progressive decisions. International cooperation appears to be quite promising in terms of improving the quality of forensic examinations and the reliability of expert opinions.

The development of international forensic standards is important for increasing the reliability, transparency and confidence in judicial evidence. Standards play an important role in the interaction of law enforcement agencies and forensic institutions of different countries during cross-border investigations, harmonizing the working methods in joint activities, facilitating the sharing of forensic results and professional information, including the exchange of databases to ensure compliance with forensic goals. Standardization of methods for collecting, analyzing, interpreting and reporting forensic data is crucial to devising a common approach to the use of evidence. This allows for the exchange of information between countries with different legal systems, aiming to achieve effective and fair justice.

## Ethics approval and consent to participate

The given manuscript is a review article. Therefore, there is no requirement for ethical approval or participation consent. This article does not contain any studies with human participants or animals performed by the authors.

## Consent for publication

The author gives full consent for the publication of the article on approval.

## Availability of data and material

All required data pertaining to the manuscript has been already provided. There is no supplemental data.

## Funding

This research received no specific grant from any funding agency in the public, commercial, or not-for-profit sectors. This research received no external funding. This study was not funded.

## Declaration of competing interest

The authors declare that they have no known competing financial interests or personal relationships that could have appeared to influence the work reported in this paper.
